# Preparation of Cellulose Nanoparticles from Foliage by Bio-Enzyme Methods

**DOI:** 10.3390/ma14164557

**Published:** 2021-08-13

**Authors:** Zhengjie Tang, Mingwei Yang, Mingli Qiang, Xiaoping Li, Jeffrey J. Morrell, Yao Yao, Yanwei Su

**Affiliations:** 1 Yunnan Key Laboratory of Wood Adhesives and Glue Products, Southwest Forestry University, Kunming 650224, China; zhengjietang@163.com (Z.T.); ymw1796233302@163.com (M.Y.); qml2889@sohu.com (M.Q.); yaoy1012@163.com (Y.Y.); domi2553@163.com (Y.S.); 2 National Centre for Timber Durability and Design Life, University of the Sunshine Coast, Locked Bag 4, Maroochydore DC, QLD 4558, Australia

**Keywords:** foliage, cellulose nanoparticles, bio-enzyme

## Abstract

There are vast reserves of foliage in nature, which is an inexhaustible precious resource. In this study, the chemical components of five foliage types (pine needles, black locust tree leaves, bamboo leaves, elm leaves and poplar leaves) were analyzed, including cellulose content, hemicellulose content, and lignin content. The bio-enzymatic method was then used to prepare cellulose nanoparticles (CNPs) from these five kinds of leaves, and the prepared CNPs were analyzed using TEM, FTIR, FESEM, and XRD. The results showed that the content of hemicellulose in bamboo leaves was the highest, and the lignin content in the other four leaves was the highest. The cellulose content in the five kinds of foliage was arranged from large to small as pine needles (20.5%), bamboo leaves (19.5%), black locust leaves (18.0%), elm leaves (17.6%), and poplar leaves (15.5%). TEM images showed that the CNPs prepared by the five kinds of foliage reached the nanometer level in width and the micrometer level in length; therefore, the CNPs prepared in this study belonged to cellulose nanofibers (CNFs). The results of FTIR and XRD showed that CNFs prepared by the enzyme treatment exhibited a typical crystalline structure of cellulose II. The degree of crystallinity (*DOC*) of CNFs prepared from pine needle, poplar leaves, and bamboo leaves are 78.46%, 77.39%, and 81.51%, respectively. FESEM results showed that the CNFs prepared from pine needles, poplar leaves and bamboo leaves by enzymatic method presents a three-dimensional (3D) network structure, and their widths are 31 nm, 36 nm, and 37 nm, respectively. This study provides a meaningful reference for broadening the use of foliage types and improving their added value.

## 1. Introduction

Foliage is the main organ of plants for photosynthesis, nutrient production, and respiration, which have a wide range of sources and massive yields. When considering the tree as an example, the trunk must grow for a certain number of years before it can be used, but the leaves will be renewed every year; even if it is not used, it will naturally wither and return to nature. At present, the main uses of foliage are as follows: making beverages (such as tea, but only a few foliage types can be used) [1], animal feed [2], industrial raw materials (such as chlorophyll extraction, eucalyptus oil extraction) [3,4,5], etc. Although many foliage types can be consumed using the aforementioned methods, they are usually only for a few specific species, which is a small part of the amount of foliage produced each year, and the vast majority of foliage is not fully used.

Due to their special properties, such as high elastic modulus, low density, high aspect ratio, nanoscale effect, and easy surface functionalization, cellulose nanoparticles (CNPs) have received extensive attention [6]. The raw material source of CNPs is abundant and can be made from wood, herbaceous plants, tunicates, algae, crab shells, bacteria, etc. [6,7,8,9]. The preparation methods of CNPs mainly include mechanical diminution (grinder, ultrasonic treatment, high-pressure homogenizer, and microfluidization), chemical diminution (sulfuric acid hydrolysis and hydrochloric acid hydrolysis), bio-enzyme methods, and combinations thereof [6].

Compared with the bio-enzyme method, the mechanical diminution will damage the cellulose and reduce the percent crystallinity of the CNPs, while chemical diminution will produce a large amount of waste liquid that is difficult to handle. Janardhnan and Sain used fungus extracted from elm trees to pretreat bleached pulp fibers (northern black spruce), resulting in a smaller diameter distribution of CNPs [10]. Henriksson successfully prepared microfibrillated cellulose nanofibers (MFC) by using a combination of enzyme treatment (endoglucanase) and mechanical shearing. MFC morphologies were better than those prepared by acid hydrolysis, and no chemical wastes were produced [11]. Paakko studied the combined use of enzyme treatment, mechanical shearing, and high-pressure homogenization to prepare nanoscale cellulose fibrils; the results showed that combining the three methods could better stratify the fiber cell wall, obtaining longer needle-like nanocellulose [12]. At present, the selection range of cellulosic raw materials for CNPs preparation by bio-enzyme methods is relatively narrow, mainly including wood and pulp fiber.

The main goals of this study were: (1) to investigate the main components of five foliage sources (pine needles, black locust tree leaves, bamboo leaves, elm leaves, and poplar leaves), including the content of cellulose, hemicellulose, and lignin; (2) to evaluate the feasibility of preparing CNPs from different leaf sources using the bio-enzyme method and assisted ultrasonic treatment. The results of this study will help improve improve the utilization of foliage, expanding raw material sources for CNPs, and exploring more environmentally friendly preparation methods for CNPs.

## 2. Materials and Methods

### 2.1. Moisture Content Measururements

The foliage in this study were pine needles (*Pinus yunnanensis* Franch.), black locust leaves (*Robinia pseudoacacia* L.), poplar leaves (*Populus* L.), elm leaves (*Ulmus pumila* L.), and bamboo leaves (*Neosinocalamus affinis* (Rendle) King f.). Approximately 1g (accurate to 0.0001 g) of foliage was wrapped in filter paper (dry to constant weight) and oven-dried at 103 ± 2 °C for 4 h. The filter paper bag was then removed and placed in a desiccator to cool for half an hour before weighing. The drying was repeated, and the sample was weighed until the quality of the filter paper bag was constant. The moisture content (*MC*) was calculated according to Equation (1):

(1) $$MC\left(\%\right)=\frac{m_{0}-m_{1}}{m_{0}}\times 100$$

where *m*_0_ is the mass of foliage before drying (g), *m*_1_ is the mass of foliage after drying (g). The final outcome is the mean value of the three measurements.

### 2.2. Lignin Content

One g of foliage (accurate to 0.0001 g) was wrapped in a qualitative filter paper and tied with absorbent cotton. The sample was then placed into a conical flask containing a 50/50 benzene-alcohol mixture (analytical grade and extracted a boiling water for 3 h. After the extraction was complete, the foliage was removed and air-dried. The benzene-alcohol extraction removed the resin, fat, tannins, and pigments which was beneficial for subsequent tests. The sample was then moved from the air-dried filter paper bag into a 150 mL conical flask with a plug along with 15 mL (72 ± 0.1) % of sulfuric acid (analytical grade) at a temperature between 12 and 15 °C. The flask was stoppered was closed to ensure that the sample was satura placed in a water bath at 18–20 °C and kept warm for 2.5 h, shaking the flask occasionally to render a more uniform reaction in the flask. The flask contents were transferred into a 1000 mL conical flask and distilled water was added to increase the total volume to 550 mL. The flask was placed in a boiling water bath for 4 h with water added during the bath to maintain total volume at 550 mL, and then allowed to cool so the acid-insoluble lignin could precipitate. The acid-insoluble lignin was filtered through quantitative filter paper washed 3 or 4 times with 3% sulfuric acid followed by hot distilled water until the liquid pH was neutral. The precipitate was air--dried to constant weight and he edge of the filter paper was monitored for neutrality with a pH test paper. The filter paper was then oven-dried to constant weight at 105 ± 2 °C. The foliage lignin content (*L*) was calculated according to Equation (2):

(2) $$L\left(\%\right)=\frac{m_{1}-m_{2}}{m_{3}}\times 100$$

where *m*_1_ is the mass of acid-insoluble lignin after drying (g), *m*_2_ is the mass of ash in the foliage (g), *m*_3_ is the mass of dry foliage (g). The samples were assessed per foliage type.

### 2.3. Holocellulose Content

Three g of foliage (accurate to 0.0001 g) was wrapped in qualitative filter paper and tied with absorbent cotton and extracted in the benzene-alcohol mixture as described in Section 2.2. The extracted foliage was then transferred into a 250 mL conical flask and 225 mL of distilled water was added, as well as 5–6 drops (0.3 mL) of glacial acetic acid and 1.5 g of sodium chlorite. The liquid was shaken well and placed in a 75 °C water bath for 1 h with frequently aggitation. Five to seven drops (0.33 mL) of glacial acetic acid (analytical grade) and 1.5 g of sodium chlorite were added and the mixture was shaken well. The mixture was maintainedat 75 °C for 1 h and this process was repeated until the sample turned white. The flask was cooled to 10 °C in a water bath and then the mixture was filtered, washed with room temperature distilled water (about 500 mL) until neutral, and then washed with acetone three times. The filter paper was oven-dried 105 ± 3 °C) to constant weight, cooled and weighed. The foliage holocellulose content (*HOC*) was calculated according to Equation (3):

(3) $$HOC\left(\%\right)=\frac{m_{4}-m_{5}}{m_{6}}\times 100$$

where *m*_4_ is the mass of holocellulose after drying (g), *m*_5_ is the mass of ash in the foliage (g), and *m*_5_ is the mass of dry foliage (g). The extractions were performed on three samples per foliage species.

### 2.4. Cellulose and Hemicellulose Content

Three g (accurate to 0.0001 g) of foliage was and placed into a 250 mL conical flask and pre-treated with benzene-alcohol extraction, glacial acetic acid, and sodium chlorite. Twenty five mL of 17.5% sodium hydroxide solution was added and stirred carefully with a flat glass rod for 2–3 min. An additional 25 mL of NaOH was added and stirred for 1 min. The sample was then poured into a conical flask and heated at 20 ± 0.5 °C for 45 min before 50 mL of 20 ± 0.5 °C distilled water was added and stirred carefully for 1–2 min. The mixture was filtered and washed three times with 25 mL of 8.3% sodium hydroxide solution. The filtrate was washed with 400 mL of 18–20 °C distilled water, soaked in 2M acetic acid at 18–20 °C for about 5 min, and then washed with water until neutral. The filter paper was oven-dried at 105 ± 3 °C to constant weight, cooled and weighed. The foliage cellulose content (*C*) was calculated according to Equation (4):

(4) $$C\left(\%\right)=\frac{m_{7}-m_{8}}{m_{9}}\times 100$$

where *m*_7_ is the mass of cellulose after drying (g), *m*_8_ is the mass of ash in the foliage (g), *m*_9_ is the mass of dry foliage (g). The procedures were performed on three samples per foliage species.

The hemicellulose content was the difference between holocellulose and cellulose content.

### 2.5. Cellulose Nano-Particle (CNP) Preparation

**Figure 1:**
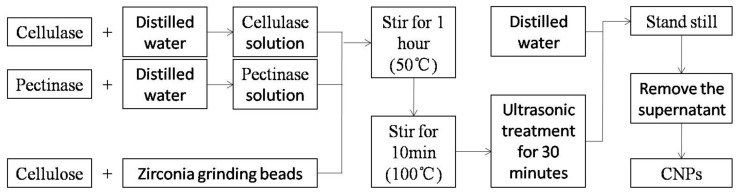
Flow chart of CNPs preparation.

Cellulase is the general name for a group of enzymes that degrade cellulose to produce glucose, a composite enzyme mainly composed of exo-β-glucanase, endo-β-glucanase, and β-glucosidase. In previous studies, long times were required to treat cellulose with cellulase [13]. In this study, combinations of cellulase and pectinase were used to treat cellulose, which considerably shortened the treatment time and improved efficiency. The flow chart of CNPs preparation is shown in Figure 1, and the detailed preparation process is as follows:

(1) Bio-enzyme solution preparation

Cellulase and pectinase solids weighing 5g were placed into a 100 mL volumetric flask, respectively, and then adding distilled water to the make-up level.

(2) CNPs preparation (Figure 1)

One g of previously prepared cellulosewas placed in a clean small beaker with 20 g of zirconia grinding beads (0.1 mm in diameter) and an magnetic stirrer. A total of 15 mL of cellulase (Aladdin Biochemical Technology Co. Ltd., Shanghai, China; cellulase activity 3000 u/mg) and pectinase (Aladdin Biochemical Technology Co. Ltd., Shanghai, China; pectinase activity 3000 u/mg) were added, respectively, and stirred for 1 h (50 °C) before being placed in a water bath at 100 °C for 10 min and subjected ultrasonic treatment for 30 min. Finally, the sample was poured into a 500 mL beaker, and diluted with 300 mL of distilled water.

### 2.6. Characterization of the CNPs

The microstructure of CNF was investigated by a transmission electron microscope (TEM, JEM 2100, Japan Electronics Co., Ltd., Tokyo, Japan) at an accelerating voltage of 120 kV. A droplet of CNF suspension (0.1 wt%) was placed onto a copper grid and dried at room temperature.

CNF powder was prepared from the CNF suspension and mixed with KBr evenly. The mixture was pressed into a thin pellet and examined using Fourier transform infrared spectroscopy (FTIR, Nicolet iS 50, Thermo Nicolet Corporation, Madison, WI, USA) to investigate the chemical structure of CNF powder. 

The morphology of CNF was examined by field emission scanning electron microscopy (FESEM, Nova NanoSEM 450, FEI Co., Ltd., Hillsboro, OR, USA). The CNF powder was placed onto the copper grid, then sputtered-coated with gold prior to examination.

X-ray diffraction (XRD, Ultima IV, Rigaku Corporation, Tokyo, Japan) was used to investigate the crystalline structure of the CNF powder with a supply of Cu-K𝛼 radiation (𝜆 = 0.154 nm). The scanning angle ranged from 5° to 60°, and the step size was 0.026° (accelerating current = 30 mA and voltage = 40 kV). The degree of crystallinity (*DOC*, %) of the CNF was calculated according to Equation (5):

(5) $$DOC\left(\%\right)=100\times \frac{I_{Max}-I_{Am}}{I_{Max}}$$

where *I_Max_* is the maximum intensity of the principal peak (around 22°), and *I_Am_* is the intensity of the diffraction attributed to amorphous cellulose (around 18°).

## 3. Results and Discussion

### 3.1. The Main Components of Foliage

The chemical content of different foliage is displayed in Table 1. The lignin content of the five kinds of foliage were arranged in the following order: black locust leaves > pine needles > bamboo leaves > poplar leaves > elm leaves. Black locust leaves had the highest lignin content (37.9%), which was 57.3% higher than Elm leaves which had the lowest lignin content (24.1%). Hemicellulose contentin the five species varied greatly. The hemicellulose content of the five kinds of foliage was as follows: bamboo leaves (37.7%), poplar leaves (22.8%), black locust leaves (21.0%), pine needles (20.3%), and elm leaves (19.6%). Cellulose content in the five species arranged from large to small was pine needles (20.5%), bamboo leaves (19.5%), black locust leaves (18.0%), elm leaves (17.6%), and poplar leaves (15.5%).

The preparation of CNPs from leaves can be divided into two steps. The first step is to purify and homogenize the leaves to obtain cellulose with high purity. The second step is to dissociate the cellulose by mechanical and chemical means to obtain nanoscale cellulose. High lignin and hemicellulose content in the leaves will negatively affect the preparation of CNPs. Lignin, as the filling material of the cell wall, will hinder separation of foliage into their component fibers; this is the reason why many studies directly choose pulp fiber as the raw material for CNPs preparation. Among the five kinds of leaves in this study, the order of preparing CNPs from easy to difficult (considered from the lignin content) should be as follows: elm leaves, poplar leaves, bamboo leaves, pine needles, and black locust leaves. The amount of cellulose in the foliage directly determines the yield of CNPs, so the order of the yield of CNPs for the five kinds of foliage is as follows: pine needles > bamboo leaves > black locust leaves > elm leaves > poplar leaves.

### 3.2. CNPs Properties

**Figure 2:**
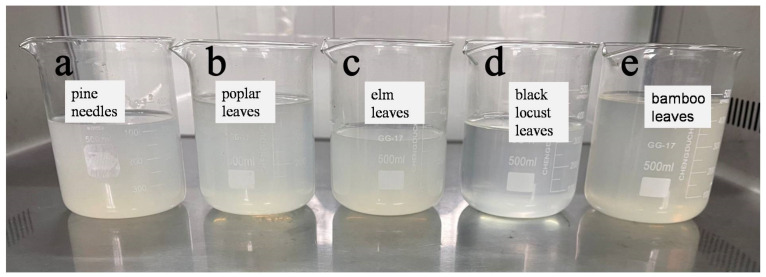
Photographs of CNPs dispersions prepared from (**a**) pine needles, (**b**) poplar leaves, (**c**) elm leaves, (**d**) black locust leaves, and (**e**) bamboo leaves.

The CNPs prepared from the five kinds of samples had relatively good stability, as shown in Figure 2. These CNPs were in a semi-transparent gel state, and there were no apparent changes, such as precipitation, after storage at room temperature for 2 weeks. In this study, the combination of cellulase and pectinase resulted in more efficient CNPs production possibly because after cellulose is degraded by cellulase, pectinase further decomposes the products obtained, improving the degradation efficiency of cellulose.

#### 3.2.1. TEM Analysis

**Figure 3:**
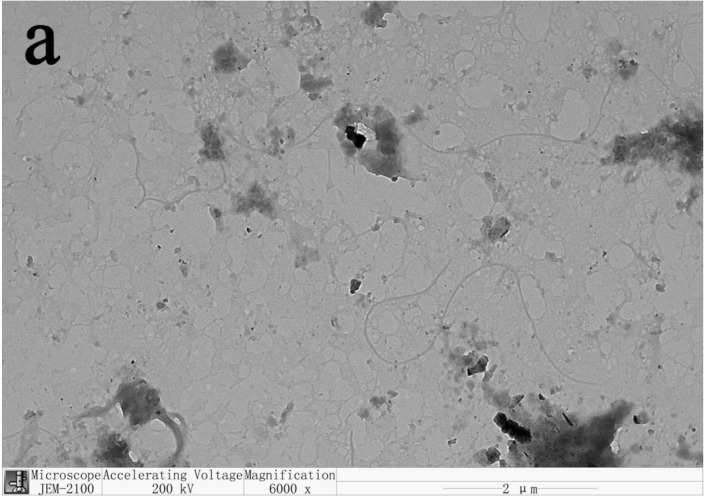

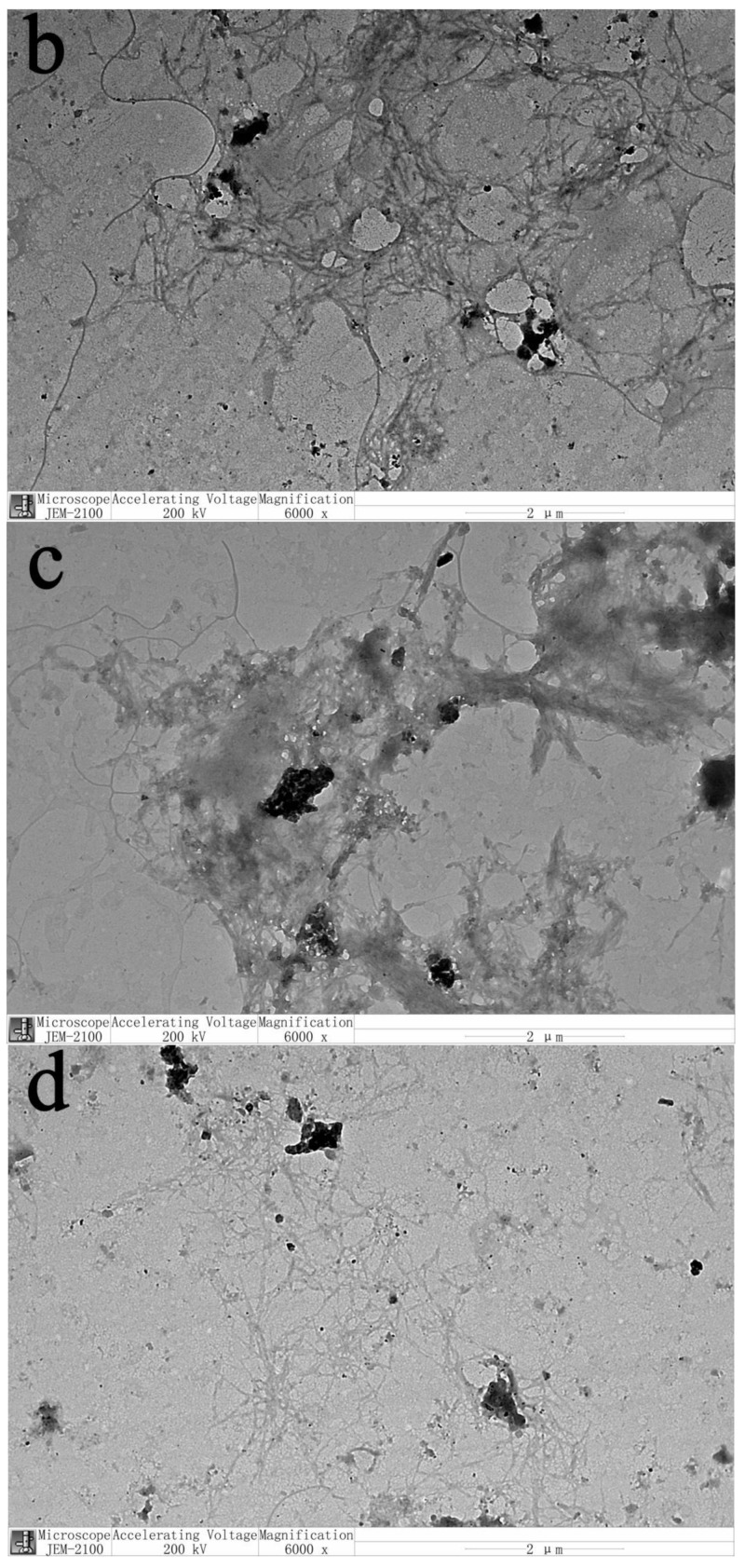

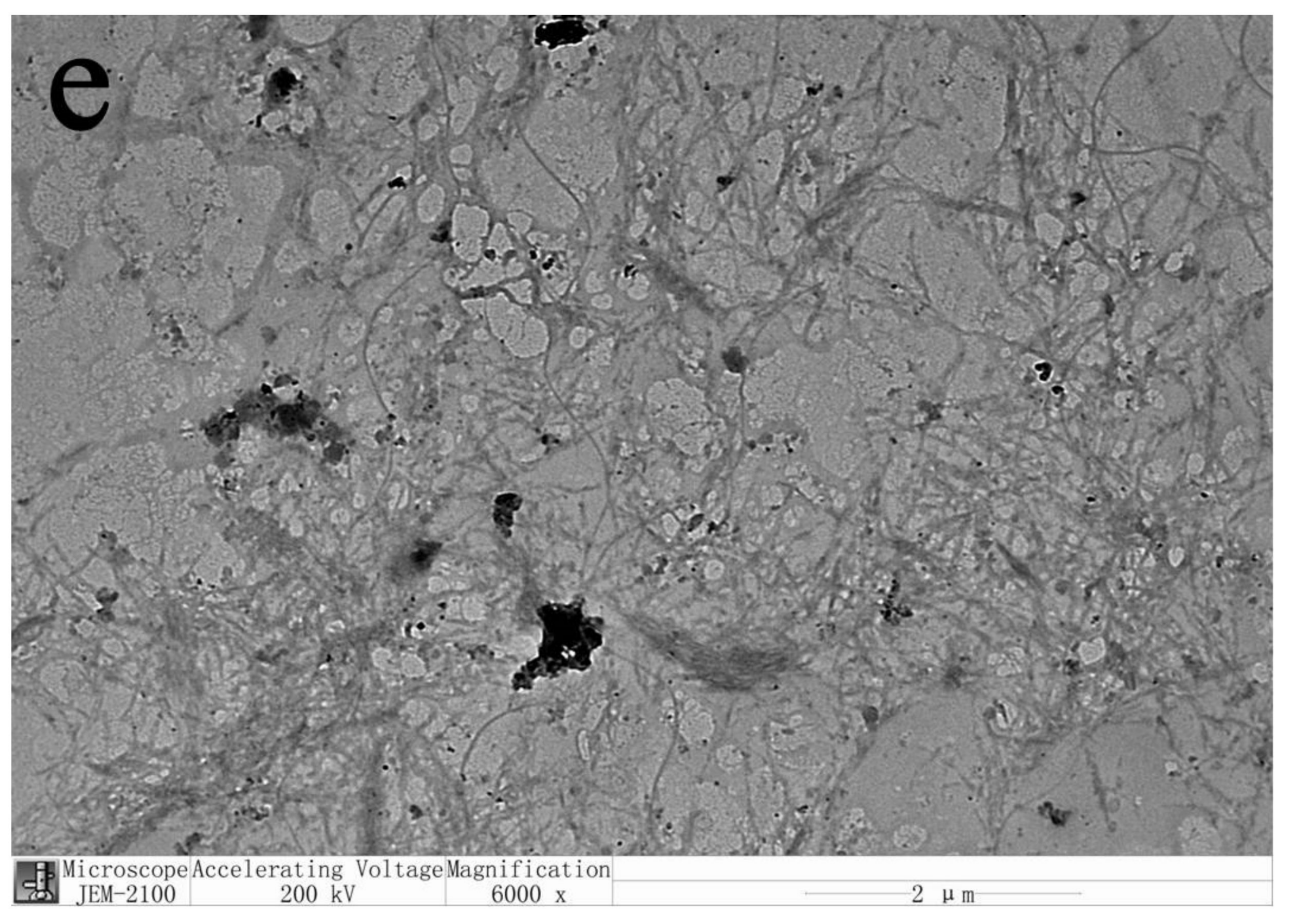
TEM micrographs of CNPs prepared from (**a**) pine needles, (**b**) black locust leaves, (**c**) poplar leaves, (**d**) elm leaves, and (**e**) bamboo leaves.

CNPs can be divided into cellulose nanofibers (CNFs) and cellulose nanocrystals (CNCs) according to their morphology and scale [14]. The transmission electron microscopic images of CNPs prepared from five kinds of leaves are shown in Figure 3. ICNPs were at the nanometer level in width and the micrometer level in length, so the CNPs prepared in this study belonged to CNFs. We can also see that due to the long length of CNFs, they are intertwined with each other, but the dispersion was relatively uniform. Therefore, the dispersion of CNFs in water was in a colloidal state and was relatively stable, as shown in Figure 1. The concentrations of CNF dispersions prepared from pine needles, black locust leaves, poplar leaves, elm leaves, and bamboo leaves were 0.259 wt%, 0.118 wt%, 0.144 wt%, 0. 129 wt%, and 0.156 wt%, respectively. The concentration of CNF affects the state of its dispersion. As the concentration increases, the CNF dispersion changed from a stable suspension to a gel state [15]. The CNF prepared in this experiment had a low concentration and presented a relatively stable suspension state, as shown in Figure 2.

#### 3.2.2. FTIR Analysis

**Figure 4:**
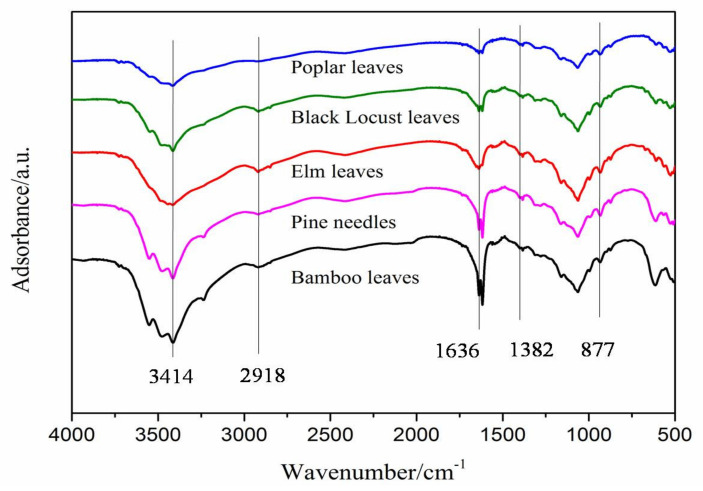
FTIR spectra of CNPs prepared from (a) pine needles, (b) black locust leaves, (c) poplar leaves, (d) elm leaves, and (e) bamboo leaves.

The FTIR signal scanned at around 3414 cm^−1^ (3500–3000 cm^−1^) was attributed to O-H stretching vibrations, and the absorption band at around 2918 cm^−1^ was attributed to C-H stretching vibrations [14]. The band at 1636 cm^−1^ was due to -OH groups of the absorbed water [15]. The peak at around 1382 cm^−1^ was attributed to C-H asymmetric deformations [15]. The signal scanned at around 877 cm^−1^ may be due to the asymmetric out-of-plane ring stretching in cellulose (the β-linkage and the amorphous form), which is more apparent in amorphous cellulose and cellulose II [16,17]. It can be seen from Figure 4 that the overall trend of the FTIR curve of CNF prepared from the five leaves is consistent. Compared with other preparation methods, in the FTIR spectrum of CNF prepared by the biological enzymatic method, a more apparent absorption peak appears at around 877 cm^−1^; These results indicated that CNF prepared by the bio-enzymatic method, not only had reduced cellulose size but also the structure of cellulose is affected.

#### 3.2.3. FESEM Analysis

**Figure 5:**
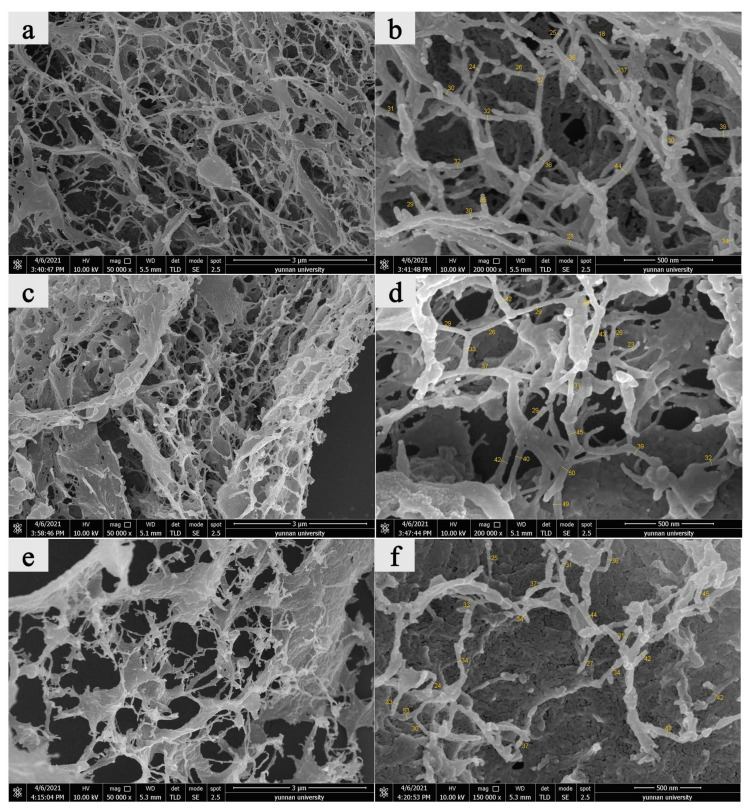
FESEM micrographs of CNPs prepared from: (**a**,**b**) pine needles, (**c**,**d**) poplar leaves, (**e,f**) bamboo leaves.

Owing to the high cellulose content in pine needles, bamboo leaves, and vast reserves of poplar leaves, the CNF prepared from the leaves of these three plants was further analyzed (FESEM and XRD) (Figure 5). CNF prepared from these three types of leaves formed an apparent 3D network structure. Therefore, adding these CNF’s to a composite as a reinforcing component should improve performance. Measurement of CNF widths from SEM micrographs showed that showed that average widths from pine needles, poplar leaves, and bamboo leaves were 31 nm, 36 nm, and 37 nm, respectively, which were all in the nanoscale range (Table 2).

#### 3.2.4. XRD Analysis

**Figure 6:**
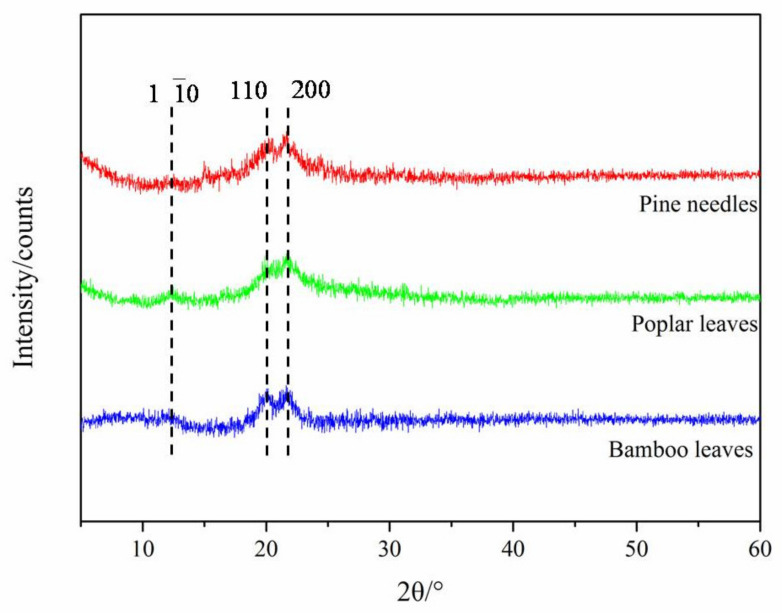
XRD spectra of CNFs prepared from pine needles, poplar leaves, and bamboo leaves.

Figure 6 shows the XRD spectra of CNF prepared from pine needle, poplar leaves, and bamboo leaves, which exhibited a crystalline structure typical for cellulose II, with the main peaks located at 12.5°, 20.2°, and 22.2°, corresponding to the crystalline planes of (1 10), (110), and (200), respectively; this is consistent with the results of the characteristic peaks in the FTIR spectra. There are six kinds of interchangeable cellulose isomers: cellulose I, cellulose type II, cellulose III _I_, cellulose III _II_, cellulose IV_I_, and cellulose IV_I_ [18]. Cellulose aggregates are widely found in the cell walls of higher plants, algae, and some bacteria with cellulose I crystal structure [1,7]. Cellulose II aggregates are currently the most widely used type of cellulose, which are obtained by chemically dissolving and regenerating or mercerizing cellulose I aggregates [18]. Unlike the cellulose crystal form of CNF prepared by mechanical grinding and chemical diminution (mainly cellulose I), CNF prepared by enzyme treatment exhibits a typical crystalline structure of cellulose II. This indicates that the preparation of CNF from leaves by the enzymatic method can transform the crystalline structure of CNF from cellulose I to cellulose II. On the other hand, the *DOC* of CNF prepared from pine needles, poplar leaves, and bamboo leaves were 78.46%, 77.39%, and 81.51%, respectively. Compared with other methods [6], the *DOC* of CNF prepared by the enzymatic method was higher, which may be due to the destruction of part of the amorphous region in the cellulose chains. Endo-β-glucanase mainly acts on the amorphous region inside the cellulose polysaccharide chain [13], thus reducing the amorphous region in cellulose, which helps explain why the crystallinity of CNPs prepared by the biological enzyme method were higher than those of CNPs prepared by other methods. The characteristics of CNF change due to the difference in cellulose structure, which would affect its potential reinforcing effect on composite materials.

## 4. Conclusions

The five foliage species evaluated in this study contain cellulose, a raw material for CNPs, with pine needles having the highest content (20.5%). The results indicated that cellulase and pectinase could be used to prepared CNFs that werein a stable colloidal state. The average widths of CNF prepared from pine needles, poplar leaves, and bamboo leaves were 31 nm, 36 nm, and 37 nm, respectively, and all three formed an apparent 3D network structure. The CNFs prepared by enzyme treatment exhibited a typical crystalline structure of cellulose II, and the *DOC* of CNFs prepared from pine needle, poplar leaves and bamboo leaves were 78.46%, 77.39%, and 81.51%, respectively. The results illustrate the feasibility of preparing CNFs from foliage using bio-enzyme methods.

## Figures and Tables

**Table 1:** Chemical compound contents of different foliage.

Chemical Compounds	Pine Needles	Black Locust Leaves	Bamboo Leaves	Elm Leaves	Poplar Leaves
MC (%)	9.01 (0.12)	9.43 (0.09)	7.82 (0.10)	10.14 (0.11)	9.14 (0.05)
Lignin (%)	29.3 (0.3)	37.9 (0.20)	25.2 (0.8)	24.1 (0.5)	25.9 (0.6)
Holocellulose (%)	40.8 (0.3)	39.0 (0.1)	57.3 (0.5)	37.1 (0.9)	38.3 (0.2)
Celluloses (%)	20.5 (0.1)	18.0 (0.6)	19.5 (0.4)	17.6 (0.2)	15.5 (0.3)
Hemicellulose (%)	20.3 (0.4)	21.0 (0.1)	37.7 (0.5)	19.6 (0.1)	22.8 (0.3)

Note: The subtraction of holocellulose and cellulose content is the hemicellulose content. Values represent means of three replicate while figures in parentheses represent one standard deviation.

**Table 2:** Fiber width of CNPs prepared from leaves/needles of three species.

Sample	Fiber Width/nm		
	Minimum Value	Maximum Value	Average Value
Pine needles	18	39	31
Poplar leaves	23	50	36
Bamboo leaves	24	53	37

## Data Availability

The data presented in this study are available on request from the corresponding author.
